# Effects of immune cells on ischemic stroke and the mediating roles of metabolites

**DOI:** 10.3389/fneur.2024.1405108

**Published:** 2024-05-28

**Authors:** Haoxiang Hu, Mi Zhou, Yunhan Zhao, Jiesheng Mao, Xiaokai Yang

**Affiliations:** Postgraduate Training Base Alliance of Wenzhou Medical University, Third Afffliated Hospital of Shanghai University (Wenzhou People’s Hospital), Wenzhou, China

**Keywords:** Mendelian randomization, ischemic stroke, IgD-CD24-AC, AA2S, metabolite, immunology

## Abstract

**Objective:**

Previous studies have not shown an association between IgD-CD24-B-cell absolute count (IgD-CD24-AC) and ischemic stroke (IS). Our study aimed to assess the causal effect of IgD-CD24-AC on IS and to explore the role of ascorbic acid 2-sulfate (AA2S) as a potential mediator.

**Methods:**

Our study was based on the largest available genome-wide association study (GWAS). Inverse variance weighting (IVW), MR–Egger, weighted median (WMN), simple mode, and weighted mode methods were used to assess causal effects, with IVW as the primary outcome. Subsequently, we further performed a two-step MR analysis to evaluate whether AA2S mediated this causal effect. In addition, several sensitivity analyses were conducted to evaluate heterogeneity, including Cochran’s Q test, the MR–Egger intercept test, the MR-PRESSO global test, and the leave-one-out analysis.

**Results:**

Using the IVW approach, the risk ratio of IgD-CD24-AC to IS was estimated to be 1.216 (95% CI = 1.079–1.371, *p* = 0.001). This result was supported by the WMN method (OR = 1.204, 95% CI = 1.020–1.421, *p* = 0.028) and the MR–Egger method (OR = 1.177, 95% CI = 0.962–1.442, *p* = 0.133). We also observed the same trend with the simple model and weighted model. Furthermore, the proportion of genetically predicted IgD-CD24-AC mediated through AA2S levels was 3.73%.

**Conclusion:**

Our study revealed a causal relationship between IgD-CD24-AC and IS, a small part of which was mediated by AA2S. These findings offer critical insights for developing immune-targeted therapies in the future and lay a strong foundation for advancements in precision medicine.

## 1 Introduction

Ischemic stroke (IS) is caused by an obstruction, generally from a blood clot, in the brain’s blood vessels (1). It is characterized not only by its high prevalence and mortality rates but also by being a significant contributor to disability and mortality among middle-aged and elderly populations, representing a formidable global health challenge (2–5). IS has been demonstrated to induce the release of inflammatory mediators, subsequently eliciting an immune response (6). IS can induce neuroinflammation characterized by impaired immune cell function, highlighting the pivotal role of immune responses in the outcomes of IS (7, 8). Furthermore, the effects of various immune cells on ischemic strokes are complex and exhibit contradictory aspects (9). B cells play an important role in this process among the various immune cells. Following an ischemic event, there is a notable accumulation of B cells in the affected area, where they actively engage in the immune response (10, 11). A previous Mendelian randomization (MR) study revealed that distinct B-cell subtypes exhibit differential impacts on IS, and this study posits that specific B-cell phenotypes, such as CD24+ B-cell phenotypes, potentially confer neuroprotection, in contrast to IgD+CD24-B cells, which may predispose individuals to a heightened risk of IS (12). Although this investigation offers preliminary insights, the intricate interplay and roles of IgD and CD24 phenotypes within the context of IS have yet to be comprehensively delineated, underscoring the critical necessity for this research.

The IgD-CD24-B-cell absolute count (IgD-CD24-AC) reflects the absolute count of B cells that are devoid of surface expression of both immunoglobulin D (IgD) and CD24 proteins. IgD, a marker protein on the surface of B cells, is usually associated with the immune response (13). CD24, an additional surface marker, is generally related to B-cell energy metabolism and selective developmental processes (14, 15). In recent years, a large number of scholars have focused on the function of the IgD and CD24 proteins on the surface of B cells in neurological diseases. Recent MR studies demonstrated that the absence of IgD protein in B cells elevates the risk for small-vessel stroke (16), with IgD protein negativity also displaying a positive correlation with the incidence of large-vessel and large-vessel atherosclerotic stroke (LAS) (17). Furthermore, an immunological analysis of individuals suffering from traumatic brain injury (TBI) revealed a universal increase in the number of IgD-CD24-B cells across all examined patients (18). In a cross-sectional study, investigators observed a significant increase in IgD- and CD24-expressing B cells among individuals diagnosed with myalgic encephalomyelitis/chronic fatigue syndrome (ME/CFS) (19). Moreover, contemporary Mendelian randomization (MR) studies have furnished compelling evidence, notably one such study illustrating that IgD+CD24-B cells significantly amplify the risk associated with the onset of Alzheimer’s disease (20). Similarly, an additional recent MR investigation revealed an increased risk for Parkinson’s disease development in the presence of IgD-CD24-B cells (21). Although these findings do not directly address the specific effects of IgD and CD24 deficiency on IS, they highlight the complexity of immune responses mediated through the IgD and CD24 proteins and their potential impact on neurological disorders.

Previous studies have shown that metabolites act as mediators that can reveal the relationship between genetic variation and disease, leading to a deeper understanding of the biological pathogenesis of human disease (22). Ascorbic acid 2-sulfate (AA2S), a phase II metabolite of ascorbic acid, is synthesized catalytically by sulfotransferases originating from the liver, exhibiting widespread distribution in the body (23), and is excreted mainly in the urine (24). Consequently, the concentration of AA2S could serve as an indicator of the metabolic processing of ascorbic acid (AA) (25). AA has a variety of physiological functions in the body, such as preventing the formation of oxygen free radicals, regulating inflammatory factors, reducing inflammatory cell infiltration, reversing endothelial dysfunction, enhancing microcirculation, and relieving microinflammatory conditions (26). Regarding the immune system, AA is a powerful first-line antioxidant with multifaceted roles. For example, it regulates the function of both innate and adaptive immune cells (27). It improves plasma cell differentiation by altering epigenetic patterns (28). It also acts as a cofactor in various biosynthetic pathways and influences redox pathways in the immune system (29, 30). Previous studies have shown that AA lowers the risk of IS by protecting the cardiovascular system against atherosclerosis through its anti-inflammatory, antioxidant, and endothelium-protective effects. In contrast, elevated levels of AA2S may suggest excessive AA depletion, which in turn increases the risk of IS (31, 32). Earlier Mendelian randomization studies revealed causal effects of AA2S levels on 26 disease traits across 12 categories in humans (33). However, this study did not identify a relationship between AA2S levels and IS. Accordingly, based on these findings, we postulate that the levels of AA2S could mediate the relationship between IgD-CD24-AC and IS.

Conventional observational epidemiological studies have numerous limitations in studying etiology and inferring causality due to confounding factors such as reverse causality and potential confounders (34). Therefore, a new design is needed to avoid or minimize these biases. Similar to randomized controlled trials (RCT), Mendelian randomization (MR) is a novel method for exploring causal relationships between exposures and outcomes (35). As a powerful statistical tool, it is used to infer causal relationships between exposures and outcomes (36). It provides a novel approach to exploring the relationship between IgD-CD24-AC and IS and its underlying mechanisms by exploiting genetic variability. The MR study design follows the Mendelian “random assignment of parental alleles to offspring,” which avoids the interference of reverse causation bias and potential confounding factors, allowing us to make more robust causal inferences (37, 38). Previous studies have assessed the causal effects of various types of biomarkers (including circulating markers, cerebrospinal fluid markers, and gut microbiota markers) on neurological diseases through MR analysis (39–41). Moreover, with the increasing abundance of genome-wide association study (GWAS) data, MR analysis methods will be a key driver of progress in the field of neurology (42). Thus, it was essential to use the MR method to explore the effects of immune cells and metabolites on IS. The objectives of our study were (i) to determine whether IgD-CD24-AC is causally associated with IS and (ii) to assess the extent to which AA2S mediates the effect of IgD-CD24-AC on IS by mediation analysis.

## 2 Methods

### 2.1 Study design

**Figure 1:**
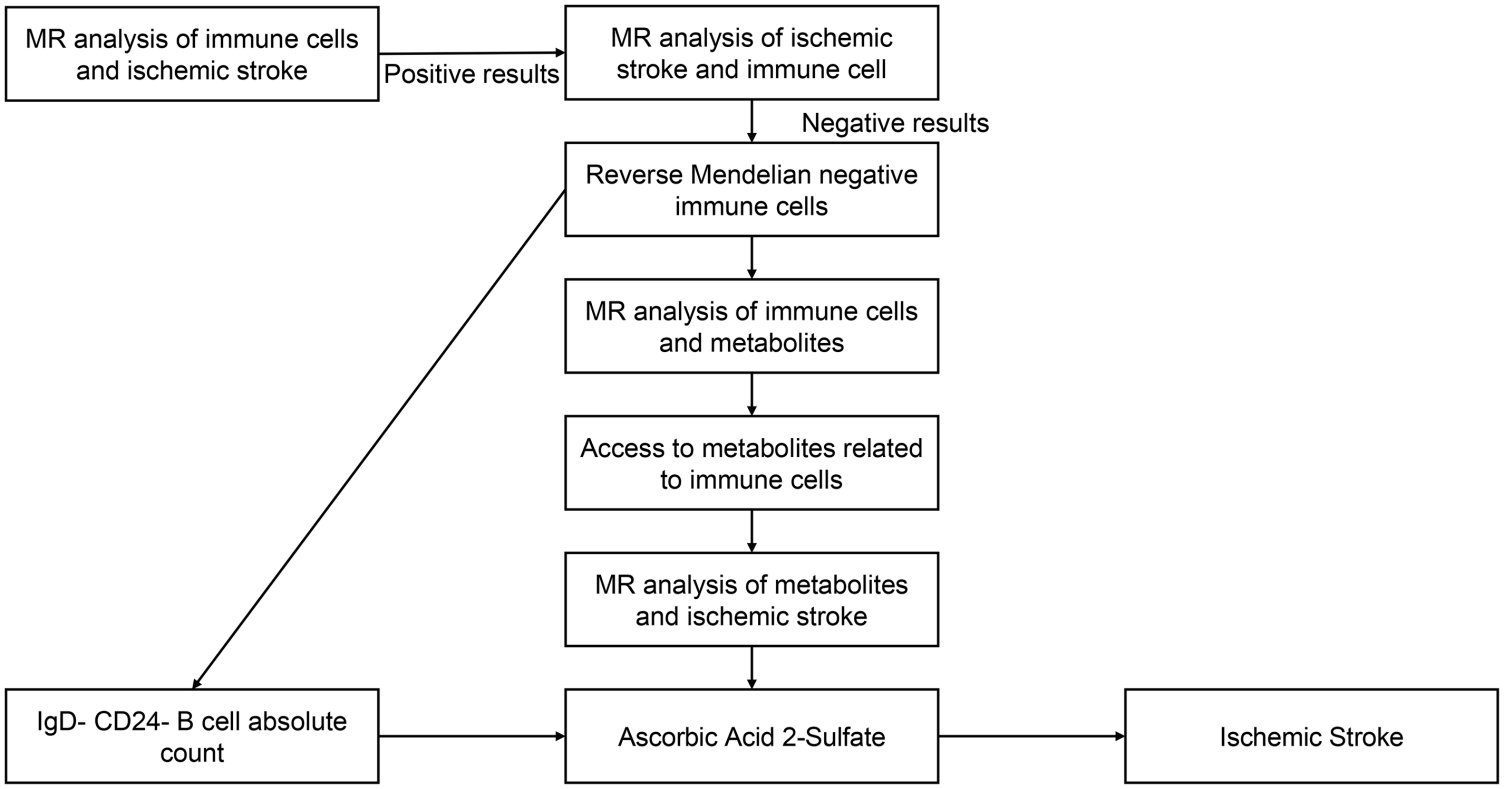
The flowchart of our MR analysis. IgD-CD24-AC, IgD-CD24-B-Cell absolute count, AA2S, Ascorbic Acid 2-Sulfate.

A convincing Mendelian randomization (MR) design must meet three basic assumptions to ensure its scientific validity and effectiveness: (1) a significant association exists between the genetic instrumental variable and the exposure variable; (2) the genetic instrumental variable is independent of confounders, and (3) the genetic instrumental variable affects the outcome only through the exposure (43). Among these assumptions, the first hypothesis represents the causal hypothesis, while the second and third hypotheses collectively refer to pleiotropy (44), which can be tested using a range of statistical methods. In our study, we initially analyzed the effects of immune cells and metabolites on IS. Subsequently, we explored the mutual causality between IgD-CD24-AC, AA2S, and IS by screening single nucleotide polymorphisms (SNPs) from 731 immune cells, 1,400 metabolites, and the FinnGen database using Mendelian randomization. These SNPs were defined as instrumental variables (IVs) (45). Figure 1 illustrates the flowchart of our analyses.

### 2.2 GWAS summary data sources

Data on IgD-CD24-AC were procured from a 2020 investigation conducted by Orrù et al. (46). The inquiry meticulously scrutinized 731 immunophenotypes, encompassing an array of parameters: 192 relative cell counts (RC), 32 morphometric attributes (MP), 118 absolute cell counts (AC), and 389 instances of median fluorescence intensity (MFI), the latter delineating the magnitude of surface antigen presence. Overall, this research revealed 122 substantial independent association signals distributed across 70 genetic loci, revealing 53 hitherto undiscovered loci and elucidating the molecules and mechanisms involved in the regulation of 459 cellular attributes. Furthermore, flow cytometry was used to determine 118 absolute cell counts (ACs), 389 median fluorescence intensities (MFIs) indicative of surface antigen levels, 32 morphological parameters (MPs), and 192 relative cell counts (RCs). The MFI indicates protein expression levels in designated cell subpopulations and reflects the median fluorescence emitted by a targeted protein. For example, the trait “IgD-CD24-B-cell” represents a negative B-cell for both IgD and CD24. These GWAS data include 3,757 nonoverlapping European individuals, including 22 million SNPs, and were tested for correlations after controlling for covariates such as age, age^2^, and sex.

Our data on AA2S come from the study by Chen (47), the most comprehensive survey of genetic loci for blood metabolites to date. Chen conducted a series of large GWASs, including the testing of 1,091 metabolites and 309 metabolite ratios in 8,299 individuals from the Canadian Longitudinal Study of Aging (CLSA) cohort. Of the 1,091 metabolites tested, 850 had known properties in eight super pathways (i.e., lipids, amino acids, xenobiotics, nucleotides, cofactors and vitamins, carbohydrates, peptides, and energy). The residual cohort of 241 molecular entities was categorized as either unknown or possessing partial characterization. These metabolites were subjected to stringent Bonferroni correction and adjusted for the total number of metabolites tested (*p* < 5 × 10^−8^/1,091 = 4.58 × 10^−11^). AA2S is metabolized via the super-pathway of vitamin C. The metabolism of AA2S was also determined by Bonferroni correction (*p* < 5 × 10^−8^/1,091 = 4.58 × 10^−11^).

To minimize sample overlap, we utilized IS data from the FinnGen consortium (Round 10)^1^ as our outcomes. A total of 374,631 controls and 1,485 patients with IS were included in our research; all of the participants were of European descent. The FinnGen study is a large-scale genomics initiative that has analyzed over 500,000 Finnish biobank samples and correlated genetic variation with health data to understand disease mechanisms and predispositions. The project involves collaboration between research organizations and biobanks in Finland and international industry partners (48).

### 2.3 Instrumental variable selection and data harmonization

For MR analyses, the genetic variants utilized must be representative of the characteristics of IgD-D24-AC and AA2S. Based on previous similar studies (49, 50), we selected single nucleotide polymorphisms (SNPs) with *p* values less than (1 × 10^−5^) as instrumental variables (IVs) to make them more informative. Furthermore, SNPs that exhibited a linkage disequilibrium (LD) coefficient (r^2^) of less than 0.001 and those positioned more than 10,000 kilobases apart were chosen to ensure independence. To quantify the strength of the IV, we calculated the *F* value using the genetic variance (R^2^), total sample size (N), and number of IVs (k) with the formula F = R^2^(N − k − 1)/k(1 − R^2^), where the genetic variance is considered weak when the F statistic is less than 10, which may bias the results (51, 52). Due to the binary nature of the outcomes being investigated, ratio estimates had to be transformed to produce the appropriate odds ratios (OR) and 95% confidence intervals (53).

### 2.4 Statistical analysis

All analyses were conducted using R version 4.3.2.^2^ The “Two Sample MR package” (54) (version 0.5.9) was used for the analyses. Additionally, the MR-Pleiotropy RESidual Sum and Outlier (MR-PRESSO) and robust adjusted profile score (MR. RAPS) methods were applied using the R packages “MRPRESSO” and “MR.raps.” Moreover, the statistical power for MR was calculated using mRnd.^3^

### 2.5 Mediation analyses

**Figure 2:**
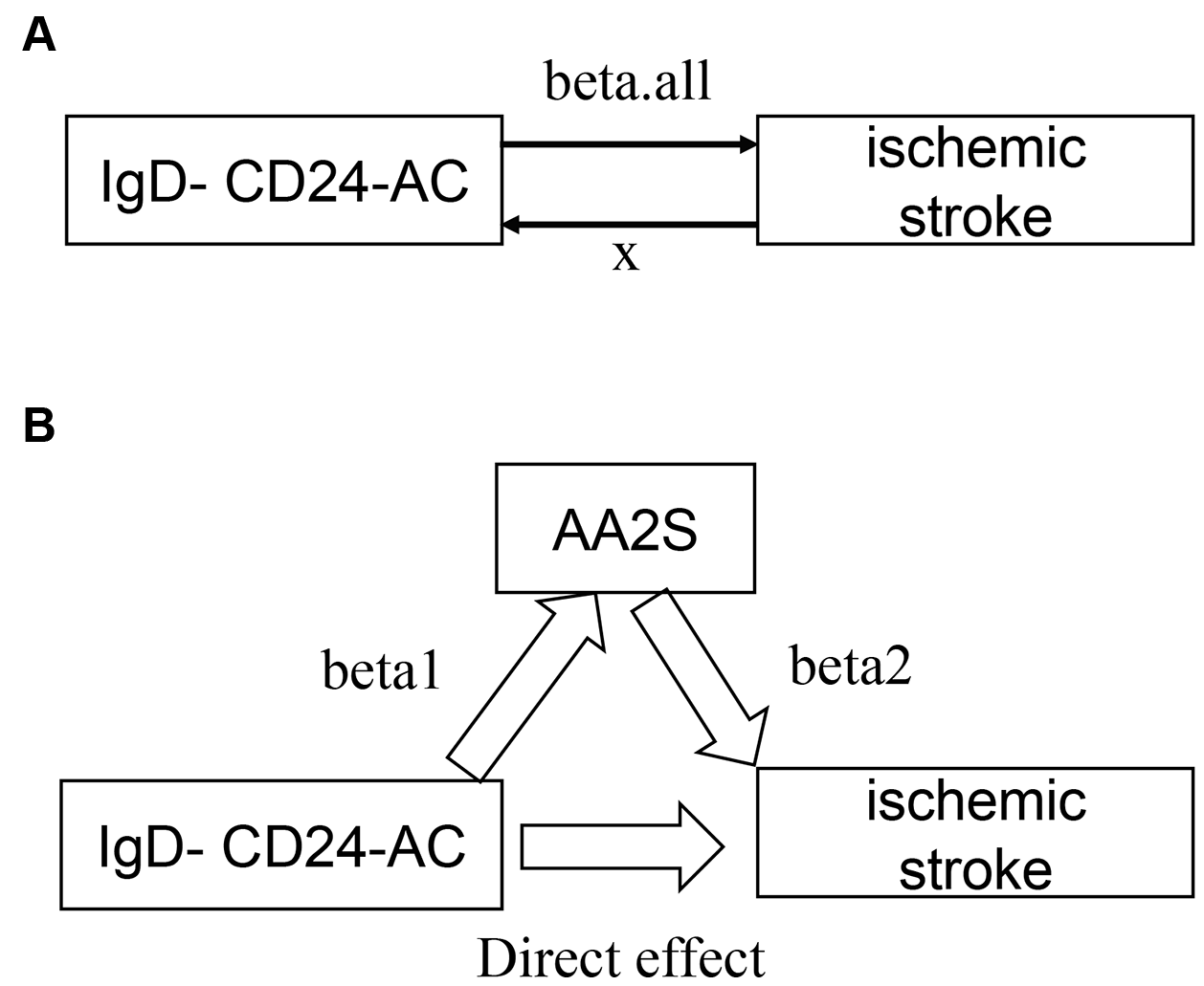
Graphs illustrate the associations examined in this study. **(A)** Total effect “beta. All” of IgD-CD24-AC with ischemic stroke. **(B)** Total effect de-composed as (i) mediated effect using a two-step approach “beta12” = beta1 × beta2 (where “beta1” is the total effect of IgD-CD24-AC on AA2S and “beta2” is the effect of AA2S on ischemic stroke effect) (ii) direct effect = “total effect” − “mediated effect” (beta. All-beta12). The mediated ratio is the indirect effect divided by the total effect. IgD-CD24-AC, IgD-CD24-B Cell absolute count, AA2S, Ascorbic Acid 2-Sulfate.

Figure 2 presents an analysis schematic. We conducted two-sample bidirectional Mendelian randomization to evaluate the reciprocal causality between IgD-CD24-AC and the IS (Figure 2A), which was referred to as the total effect (beta. all).

The causal relationship between IgD-CD24-AC and IS was assessed through a suite of methods: inverse variance weighting (IVW) (35), weighted median (WMN) (55), MR-Egger (56), and simple model and weighted model (57). Employing the IVW approach, which aggregates Wald ratio estimates of each instrumental SNP through a meta-analysis-like methodology, allowed for precise effect estimation. The results are reported as beta values with 95% confidence intervals (CIs) for standard errors and odds ratios (ORs) for continuous outcomes. For binary outcomes, *p* < 0.05 was considered nominally significant. The MR–Egger method, complemented by weighted median, weighted modal, and simple modal analyses, was used to assess multivariate validity as a Supplementary Method to bolster the reliability of IVW findings. A principal advantage of the weighted median approach lies in its ability to yield consistent causality estimates, even when more than 50% of the instrumental variables are invalidated (55).

An investigation into whether AA2S mediated the causal pathway from IgD-CD24-AC to IS outcomes (Figure 2B), which was involved in conducting mediation analyses, was performed using a two-step MR. The overall effect can be broken down into indirect (mediated) and direct effects (58). Therefore, the impact of IgD-CD24-AC on IS was divided into two components: (1) the direct effect of IgD-CD24-AC on IS (direct effect in Figure 2B) and (2) the indirect effect of IgD-CD24-AC on IS mediated by AA2S (beta1 × beta2 in Figure 2B). Subsequently, we calculated the percentage of mediated effects by dividing the indirect effect by the total effect. Additionally, we used the delta method to calculate 95% confidence intervals (59).

### 2.6 Sensitivity analyses

Heterogeneity markers from the IVW and MR–Egger approaches (Cochran Q-derived *p* < 0.05) were utilized as indicators of potential pleiotropy (60–62). By contrasting the Egger intercept term with the null result, MR–Egger analysis was able to evaluate directional pleiotropy (55). In addition, the MR-PRESSO test was used to assess the total pleiotropy of the studies and to identify any SNPs that were unusual or showed horizontal pleiotropy. To ensure the robustness of the effects, the MR analysis included an assessment before and after the removal of outlier SNPs (56, 62). If an outlier SNP was detected (*p* < 0.05), it was excluded, and the causal and sensitivity analyses were reconducted using a random-effects model to ensure the reliability of the results.

## 3 Results

### 3.1 Primary results

Among 731 immune cells and 1,400 metabolites, 30 immune cell features were initially identified as being associated with IS by the IVW method (Supplementary Figure S1), as well as 36 metabolites (Supplementary Figure S2). After performing a two-step MR analysis (Figure 1) and sensitivity analyses, it was finally determined that IgD-CD24-AC was associated with IS and that AA2S acted as its mediator.

### 3.2 Association of IgD-CD24-AC with IS

**Figure 3:**
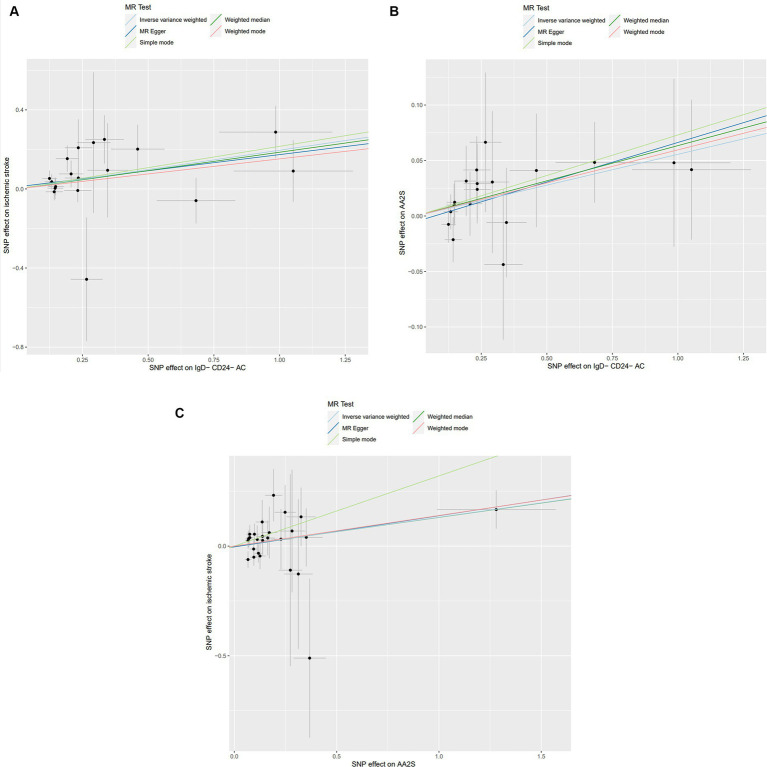
Scatter plot of genetic association between IgD-CD24-AC, AA2S, and IS. **(A)** Association of IgD-CD24-AC with IS; **(B)** Association of IgD-CD24-AC with AA2S; **(C)** Association of AA2S with IS. The slope and direction of the straight line represent the magnitude and direction of the causal relationship. IgD-CD24-AC, IgD-CD24-B Cell absolute count, AA2S, Ascorbic Acid 2-Sulfate.

**Figure 4:**
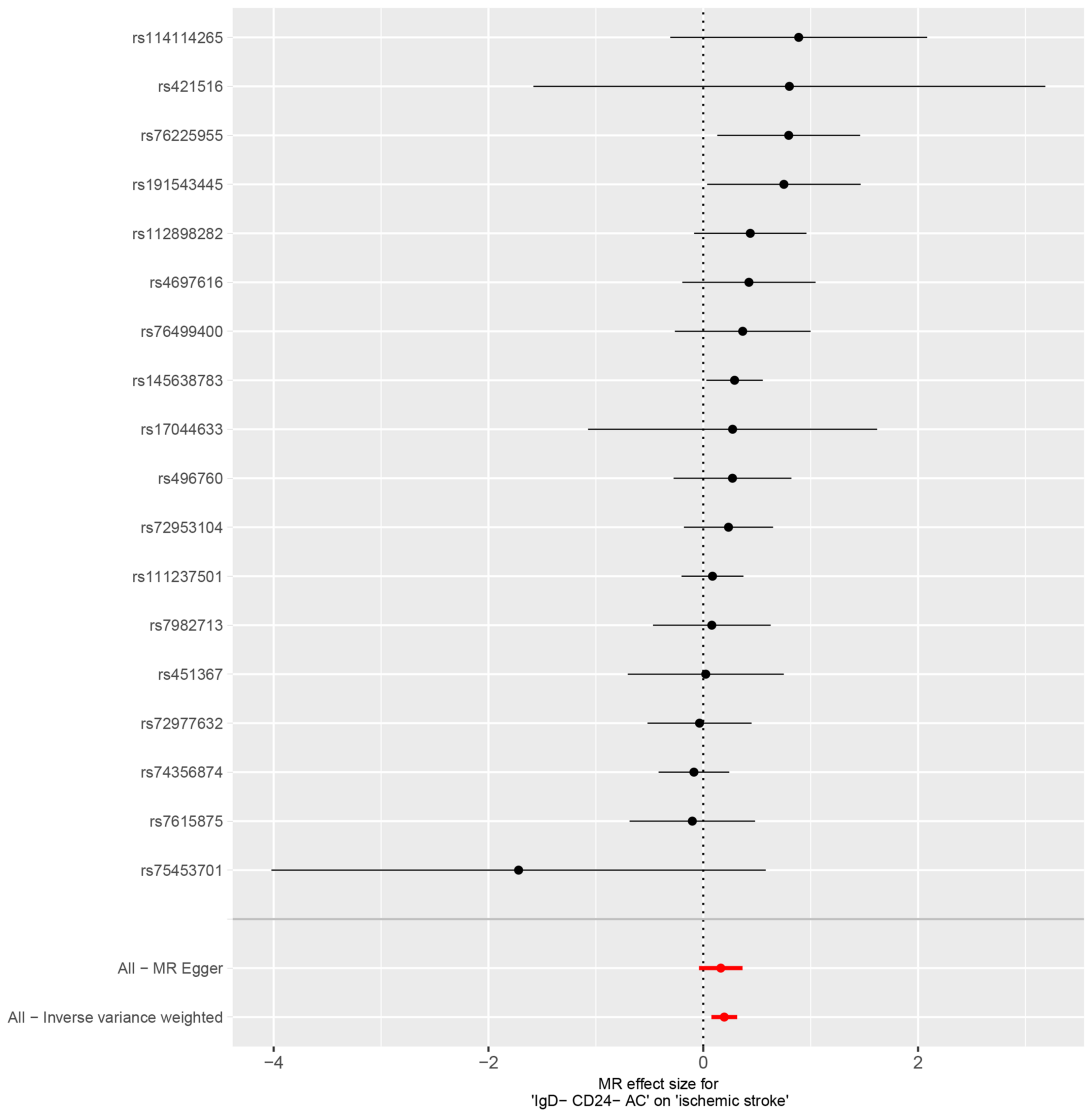
Forest plot of genetic association between IgD-CD24-AC and IS.

**Figure 5:**
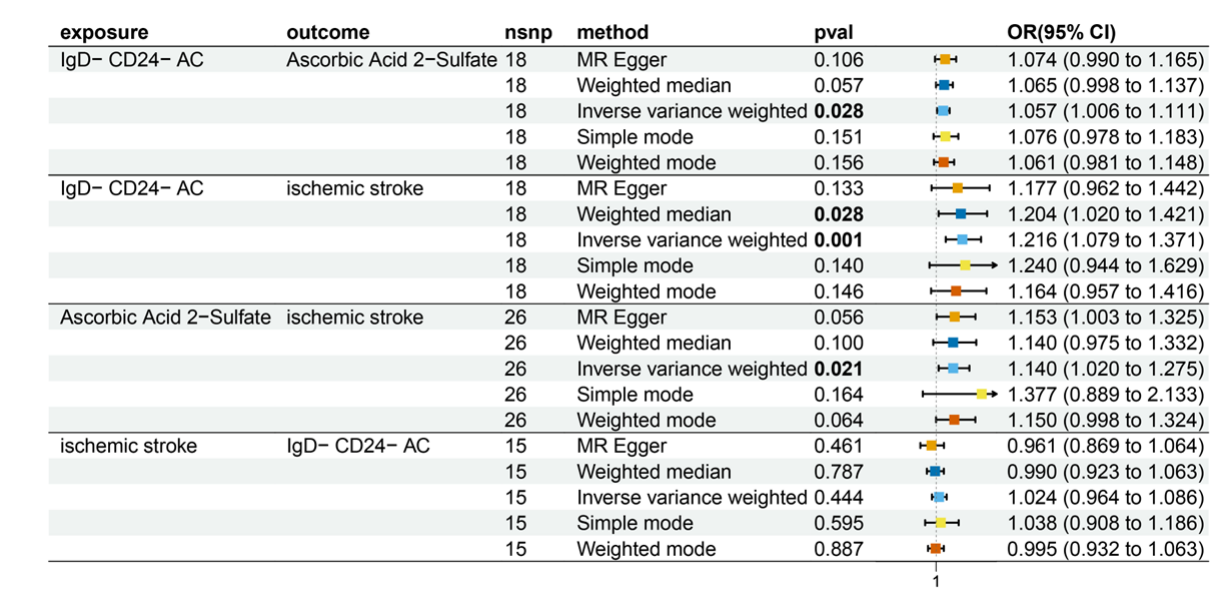
Forest plot to visualize the causal effects of AA2S with IgD-CD24-AC and ischemic stroke. IgD-CD24-AC, IgD-CD24-B Cell absolute count, AA2S, Ascorbic Acid 2-Sulfate.

Characteristics of significant SNPs with genome-wide associations in Supplementary Table S2. All SNPs used to measure IgD-CD24-AC exposure had *F* values greater than 10. We identified 18 SNPs as instrumental variables. Our results revealed a causal effect of IgD-CD24-AC on IS. As shown in Figures 3A, 4, the positive correlation between IgD-CD24-AC and the IS was broadly and consistently demonstrated across all five MR analysis methods. Employing the IVW approach, the risk ratio of IgD-CD24-AC to IS was estimated to be 1.216 (95% CI = 1.079–1.371, *p* = 0.001). This result was supported by both the weighted median method (OR = 1.204, 95% CI = 1.020–1.421, *p* = 0.028) and the MR–Egger method (OR = 1.177, 95% CI = 0.962–1.442, *p* = 0.133). The same trend was also observed in the simple and weighted models. The detailed results are displayed in Figure 5.

### 3.3 Association of IgD-CD24-AC with AA2S

Supplementary Table S4 contains Characteristics of significant SNPs with genome-wide associations and the F-statistics for all SNPs utilized in MR studies that were larger than 10. Figure 3B shows that genetically predicted IgD-CD24-AC was positively linked and trended consistently with AA2S risk using the five MR analysis methods., we discovered that genetically predicted IgD-CD24-AC was positively linked and trended consistently with AA2S risk using the five MR analysis methods. Specifically, the IVW method estimated the ratio of initial IgD-CD24-AC to IS risk to be 1.057 (95% CI = 1.006–1.111, *p* = 0.028). The weighted median (OR = 1.065, 95% CI = 0.998–1.137, *p* = 0.057) and MR–Egger (OR = 1.074, 95% CI = 0.990–1.165, *p* = 0.106) estimates were consistent. The detailed results are shown in Figure 5.

### 3.4 Association of AA2S with IS

The SNP data can be found in Supplementary Table S5. Every genetic tool linked to AA2S is listed here at the genome-wide significance level (*p* < 5 × 10^−5^). The results from the genetic prediction using the IVW method showed a strong positive association between AA2S and IS (OR = 1.140, 95% CI, 1.020–1.275; *p* = 0.021). As shown in Figure 3C, all four of the remaining methods had consistent estimation directions. Figure 5 shows the detailed results.

### 3.5 Proportion of the association between IgD-CD24-AC and IS mediated by AA2S

**Figure 6:**
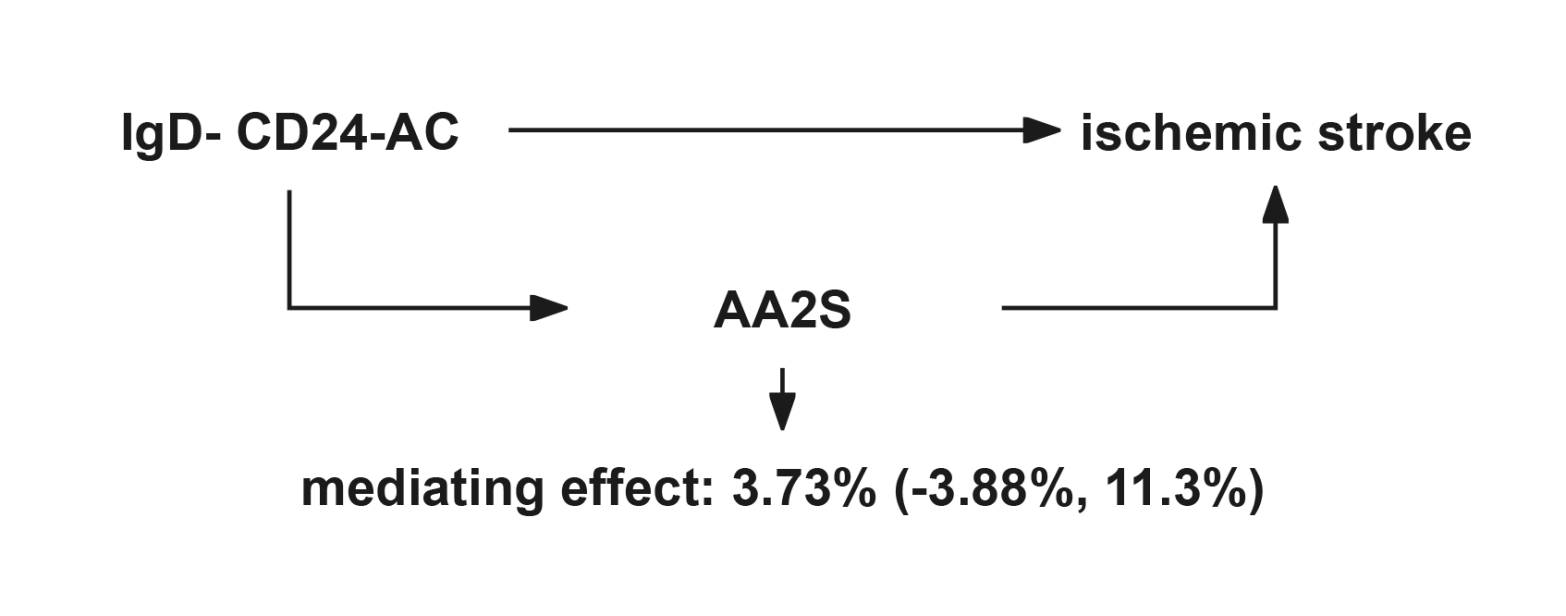
Schematic diagram of the AA2S mediation effect. IgD-CD24-AC, IgD-CD24-B Cell absolute count, AA2S, Ascorbic Acid 2-Sulfate.

The link between IgD-CD24-AC and the IS is mediated by AA2S. According to our research, AA2S was responsible for 3.73% of the elevated risk of IS linked to IgD-CD24-AC (mediator ratio: 3.73%; −3.88, 11.3%). Figure 6 displays the results.

### 3.6 Sensitivity analysis

We performed a series of sensitivity analyses to identify and address the existence of heterogeneity and pleiotropy in causality estimation. Neither the Cochran’s Q test nor the MR-Egger intercept showed evidence of heterogeneity in causality between these SNPs. We assessed potential horizontal pleiotropy in our study, which showed no horizontal pleiotropy. In addition, no influential SNPs were detected in the “leave-one-out” analysis when any of the SNPs were excluded sequentially. The distribution of the funnel plots was also symmetrical. These results indicate that all SNPs were significant for causality and that there was no heterogeneity or pleiotropy (Supplementary Table S6; Supplementary Figures S3, S4).

## 4 Discussion

In this study, we primarily identified 30 immune cells and 36 metabolites associated with IS. Then we used available genome-wide association study (GWAS) data with MR analysis to investigate the association between IgD-CD24-AC and IS and examined whether this causality was mediated through AA2S. The results showed that genetically predicted IgD-CD24-AC levels were significantly associated with an increased risk of IS (a 21.6% increase in AA2S risk for every 1 SD increase in IgD-CD24-AC) and that 3.73% of this effect was mediated through AA2S. This study represents a pioneering effort to systematically delineate the causal relationship between IgD-CD24-AC and IS and to confirm the role of AA2S as a mediator.

One of the vital pathological mechanisms of acute IS is the dysregulation of inflammatory and adaptive immune responses (63, 64). The immune response is frequently associated with oxidative stress (65), which is a state of cellular damage resulting from the overproduction of reactive oxygen species (ROS). When ROS concentrations exceed the ability of antioxidants to maintain redox balance, cellular and vascular damage occurs (66). Abnormalities in energy metabolism and brain tissue infarction caused by IS increase ROS levels in the affected area, causing the release of inflammatory factors, which stimulate an immune response (67–69). Previous studies have suggested that a compromised blood–brain barrier after an IS may facilitate the entry of B cells and other peripheral immune cells into damaged brain tissue (10, 11). However, the specific effects of the absence of IgD and CD24 proteins on the surface of B cells on IS and the underlying mechanisms are unclear, suggesting the need to explore further the nuanced roles of IgD and CD24 in IS. Previous research has indicated that CD24 functions as a growth-promoting factor and can trigger adenosine monophosphate kinase (AMPK) activation via phosphorylation, which is one of the critical signals in the overall metabolic pathway (70). Cells can activate this pathway under hypoxic conditions to boost ATP production and maintain energy balance. Furthermore, research has shown that when this enzyme is deactivated, CD24 expression decreases (15). IgD is crucial for regulating both innate and adaptive immune responses (13). If IgD is not present on the B-cell surface, it can lead to maladaptive immunity. Research has demonstrated that dysfunctional immune processes can result in a greater likelihood of experiencing stroke (63). In addition, IgD transmits regulatory signals at the B-cell receptor (BCR) to promote the formation of protective immunoglobulins, which helps to prevent autoimmune reactions and prevent early apoptosis (71). Studies in mouse models have highlighted the role of IgD in preventing premature differentiation of B cells into short-lived plasma cells (72). Thus, CD24 is crucial for B-cell selection and development (14). B cells that do not express CD24 and IgD not only have a weaker immune response but also a shorter lifespan. This study confirmed that the risk of IS increases with an increase in the number of IgD-CD24-B cells. One possible mechanism is the impaired energy metabolism of immune cells and reduced immune response capacity due to the absence of CD24 and IgD, which consequently elevates the risk of IS.

The strength of our study lies in the utilization of comprehensive, up-to-date data on 731 immune cells, 1,400 metabolites, and various IS datasets from multiple databases. Through magnetic resonance analysis, we hypothesize that the presence of IgD and CD24 on B immune cells elevates the risk of IS. AA2S, a metabolite of AA, has been shown in early animal experiments to possess weaker antioxidant properties compared to AA (73). Some experiments suggest that AA may mediate the interaction between immune cells and disease through complex mechanisms (31, 32). However, research on the correlation between AA2S and immune cells remains limited. Our findings indicate that the antioxidant effect of AA2S diminishes as its levels increase, thus elevating the risk of IS. This aligns with the correlation between IgD-CD24-AC and IS. Further mediation analyses have demonstrated that AA2S leads to increased levels of IgD-CD24-AC, heightening the risk of IS by 3.73%. Although the percentage is relatively small and other mediators that have not yet been investigated may also have a role, this is still clinically significant. This study provides theoretical support for strategies to prevent, reverse, and mitigate IS. It also highlights the dual role of AA2S in increasing IS risk, both directly and mediated by IgD-CD24-AC. These insights are crucial for the development of targeted immune therapies and for establishing a solid foundation for the advancement of precision medicine.

There are several limitations in this study. Our findings should be interpreted cautiously when applying them to ethnicities other than European populations due to the limited SNP data available. Second, despite our attempts to identify and remove abnormal variations, we cannot entirely exclude the potential influence of pleiotropy on our findings. In our study, we utilized summary-level statistics instead of individual-level data. Hence, we could not investigate causal relationships between subgroups such as females and males in more depth. The genetic prediction rate of IS mediated by AA2S levels in our study was 3.73%, indicating a very low level of prediction. More research is needed to investigate and measure more potential mediators in the future.

## 5 Conclusion

Our study identified a causal relationship between the Absolute Count of IgD-CD24-B cells and IS, a small part of which is mediated by AA2S levels. These findings offer critical insights for developing immune-targeted therapies in the future and lay a strong foundation for advancements in precision medicine.

## Data availability statement

The datasets presented in this study can be found in online repositories. The names of the repository/repositories and accession number(s) can be found in the article/Supplementary material.

## Ethics statement

Ethical review and approval was not required for the study on human participants in accordance with the local legislation and institutional requirements. Written informed consent from the patients/participants or patients/participants' legal guardian/next of kin was not required to participate in this study in accordance with the national legislation and the institutional requirements.

## Author contributions

HH: Conceptualization, Data curation, Formal analysis, Writing – original draft, Writing – review & editing. MZ: Conceptualization, Data curation, Writing – original draft. YZ: Conceptualization, Writing – original draft. JM: Conceptualization, Data curation, Writing – original draft, Writing – review & editing. XY: Conceptualization, Data curation, Methodology, Writing – original draft, Writing – review & editing.

## Footnotes

^1^ https://www.finngen.fi/en

^2^ https://www.r-project.org/

^3^ https://cnsgenomics.shinyapps.io/mRnd/
