# Supplementary material for: Effects of immune cells on ischemic stroke and the mediating roles of metabolites
Source: Front Neurol. 2024 May 28;15:1405108. doi: 10.3389/fneur.2024.1405108 (PMC11165215; doi:10.3389/fneur.2024.1405108)

**INDEX**	
**Supplementary Table S1**	Details of the data sources used in this study
**Supplementary Table S2**	Characteristics of significant SNPs with genome-wide associations (P<5×10-5) for IgD- CD24- AC on IS
**Supplementary Table S3**	Characteristics of significant SNPs with genome-wide associations (P<5×10-5) for IS on IgD- CD24- AC
**Supplementary Table S4**	Characteristics of significant SNPs with genome-wide associations (P<5×10-5) for IgD- CD24- AC onAA2S
**Supplementary Table S5**	Characteristics of significant SNPs with genome-wide associations (P<5×10-5) for AA2S on IS
**Supplementary Table S6**	Heterogeneity and pleiotropy in MR analyses.
**Supplementary Figure S1**	Forest plot of causal effect of immune cells on IS
**Supplementary Figure S2**	Forest plot of causal effect of metabolites on IS
**Supplementary Figure S3**	leave one out sensitivity analysis for IgD- D24- AC on IS
**Supplementary Figure S4**	funnel plot of IgD- D24- AC on IS
**Supplementary Figure S5**	Forest plot of causal effect of AA2S on total IS risk
**Supplementary Figure S6**	Forest plot of causal effect of IgD- D24- AC on AA2S

Supplementary Table S1: Details of the data sources used in this study			
**Exposure or Outcome**	**Consortium**	**Ancestry**	**Sample**
IgD- CD24- B cell absolute count	Valeria Orrù's study	European	3656 cases
Ascorbic Acid 2-Sulfate	Chen's study	European	8089 cases
Ischemic Stroke	finngneR10	European	1485 cases and 374631 controls

Supplementary Table S2: Characteristics of significant SNPs with genome-wide associations (P<5×10-5) for IgD- CD24- AC on IS

SNPs	CHR	POS	EA	OA	BETA	SE	P	F
rs111237501	1	163302911	C	T	1.052	0.2261	3.35E-06	21.64
rs112898282	10	28173255	C	T	0.4601	0.1017	6.28E-06	20.46
rs114114265	2	79579586	A	G	0.2343	0.05149	5.51E-06	20.69
rs145638783	6	1238207	A	G	-0.9851	0.2144	4.47E-06	21.10
rs17044633	4	112410492	G	A	0.3458	0.07605	5.62E-06	20.66
rs191543445	14	31035932	G	A	0.3337	0.07236	4.14E-06	21.26
rs421516	17	65139471	A	C	-0.2921	0.06361	4.54E-06	21.08
rs451367	19	16302031	C	T	-0.146	0.0296	8.52E-07	24.32
rs4697616	4	25765561	A	G	0.1246	0.02682	3.53E-06	21.57
rs496760	15	54403488	A	G	0.1331	0.02656	5.69E-07	25.10
rs72953104	1	40913911	G	C	0.2344	0.05087	4.2E-06	21.22
rs72977632	19	1930175	A	T	-0.2323	0.05115	5.75E-06	20.61
rs74356874	5	22936453	A	G	0.6821	0.1488	4.74E-06	21.00
rs75453701	13	54655511	G	A	0.2658	0.05994	9.47E-06	19.65
rs7615875	3	29303920	C	T	0.1425	0.03201	8.85E-06	19.81
rs76225955	4	31592382	C	T	-0.1924	0.0432	8.66E-06	19.82
rs76499400	3	192170840	T	G	0.2069	0.04589	6.77E-06	20.32
rs7982713	13	70802190	G	A	-0.1482	0.03033	1.07E-06	23.86

IgD- CD24- AC,IgD- CD24- B Cell absolute count

CHR, chromosome; POS, position; EA, effect allele; OA, other allele; EAF, effect allele frequency.

Supplementary Table S3: Characteristics of significant SNPs with genome-wide associations (P<5×10-5) for IS on IgD- CD24- AC

SNPs	CHR	POS	EA	OA	BETA	SE	P	F
rs11593506	10	20332488	C	T	-0.302034	0.0635888	2.04E-06	22.56
rs117489926	12	94222991	A	G	-0.445033	0.1007	9.90E-06	19.53
rs140567609	7	141993832	A	G	-1.74217	0.385863	6.33E-06	20.39
rs147653723	3	77493	C	G	-0.438687	0.096845	5.90E-06	20.52
rs148713750	7	14930668	G	A	2.10427	0.451385	3.13E-06	21.73
rs17055944	5	158029469	T	G	0.328383	0.0725319	5.97E-06	20.50
rs17133760	11	74870295	C	T	-0.481669	0.103845	3.51E-06	21.51
rs1800949	6	132128564	T	C	0.21429	0.0464028	3.87E-06	21.33
rs4129315	18	22683434	G	A	0.292548	0.0639168	4.72E-06	20.95
rs450060	5	94567371	C	T	-0.257058	0.0547026	2.61E-06	22.08
rs643101	6	101726103	T	G	-0.210854	0.0461835	4.98E-06	20.84
rs72740163	15	55366465	G	T	-1.08487	0.218177	6.61E-07	24.72
rs7355146	1	201988112	C	T	0.192265	0.0415102	3.63E-06	21.45
rs7479665	11	11723349	C	A	0.254717	0.0570048	7.88E-06	19.97
rs764596	12	115921838	G	A	-0.63491	0.137203	3.70E-06	21.41

IgD- CD24- AC,IgD- CD24- B Cell absolute count

CHR, chromosome; POS, position; EA, effect allele; OA, other allele; EAF, effect allele frequency.

Supplementary Table S4: Characteristics of significant SNPs with genome-wide associations (P<5×10-5) for IgD- CD24- AC onAA2S

SNPs	CHR	POS	EA	OA	BETA	SE	P	F
rs111237501	1	163272701	C	T	1.052	0.2261	3.34703E-06	21.64
rs112898282	10	28462184	C	T	0.4601	0.1017	6.27798E-06	20.46
rs114114265	2	79806712	A	G	0.2343	0.05149	5.50896E-06	20.69
rs145638783	6	1238442	A	G	-0.9851	0.2144	0.000004469	21.10
rs17044633	4	113331648	G	A	0.3458	0.07605	5.62406E-06	20.66
rs191543445	14	31505138	G	A	0.3337	0.07236	4.14295E-06	21.26
rs421516	17	63135589	A	C	-0.2921	0.06361	4.54496E-06	21.08
rs451367	19	16412842	C	T	-0.146	0.0296	8.51805E-07	24.32
rs4697616	4	25767183	A	G	0.1246	0.02682	3.53403E-06	21.57
rs496760	15	54695686	A	G	0.1331	0.02656	5.69102E-07	25.10
rs72953104	1	41379583	G	C	0.2344	0.05087	4.19701E-06	21.22
rs72977632	19	1930174	A	T	-0.2323	0.05115	5.75294E-06	20.61
rs74356874	5	22936562	A	G	0.6821	0.1488	4.73598E-06	21.00
rs75453701	13	55229646	G	A	0.2658	0.05994	9.47305E-06	19.65
rs7615875	3	29345411	C	T	0.1425	0.03201	8.8481E-06	19.81
rs76225955	4	31594004	C	T	-0.1924	0.0432	8.65506E-06	19.82
rs76499400	3	191888629	T	G	0.2069	0.04589	6.76893E-06	20.32
rs7982713	13	71376322	G	A	-0.1482	0.03033	0.000001068	23.86

IgD- CD24- AC,IgD- CD24- B Cell absolute count，AA2S,Ascorbic Acid 2-Sulfate

CHR, chromosome; POS, position; EA, effect allele; OA, other allele; EAF, effect allele frequency.

Supplementary Table S5: Characteristics of significant SNPs with genome-wide associations (P<5×10-5) for AA2S on IS

SNPs	CHR	POS	EA	OA	BETA	SE	P	F
rs10214728	6	148266946	T	C	-0.138442	0.0306455	6.25647E-06	20.40
rs10509168	10	62498069	C	T	0.0668528	0.0151146	9.73077E-06	19.56
rs114665461	6	38015817	A	G	-0.170847	0.0384091	8.66344E-06	19.78
rs115475653	6	36634902	G	A	-0.248047	0.0511614	1.24507E-06	23.50
rs12414218	10	87722022	A	G	-0.0953696	0.0158215	1.66174E-09	36.33
rs13227023	7	86563323	A	G	-0.125571	0.0267413	2.65629E-06	22.04
rs139448719	17	75121666	A	G	0.163133	0.0338571	1.44796E-06	23.21
rs139754472	3	140721229	T	C	0.367963	0.0774874	2.0475E-06	22.54
rs140229512	1	73425966	T	A	0.137821	0.031189	9.92008E-06	19.52
rs1405100	4	82697049	A	C	-0.0981967	0.0214383	4.64025E-06	20.98
rs1468626	15	96948385	C	T	-0.0753816	0.0158605	2.00635E-06	22.58
rs147620705	5	141885330	C	T	0.312878	0.0686197	5.12534E-06	20.78
rs2016106	2	239510106	A	G	-0.0755901	0.0156852	1.44146E-06	23.22
rs2336850	11	17709937	A	G	-0.282079	0.0629438	7.41403E-06	20.08
rs2347803	1	164909643	T	C	0.0669031	0.0150715	9.03582E-06	19.70
rs34049928	16	29971694	G	C	0.191647	0.0410303	2.9995E-06	21.81
rs35956182	6	160143584	A	G	-0.351534	0.078237	7.01645E-06	20.18
rs3765159	10	2500353	A	G	-1.27989	0.289217	9.62746E-06	19.58
rs4294667	13	107368585	T	G	-0.0944892	0.0201498	2.74098E-06	21.98
rs513107	18	66584353	G	A	-0.0701522	0.0158456	9.54464E-06	19.60
rs73143004	12	99287930	C	A	0.136203	0.0308193	9.8966E-06	19.53
rs75017029	16	74159313	C	T	-0.227788	0.0490354	3.39453E-06	21.57
rs75142236	2	29497963	T	C	0.111935	0.0238475	2.68218E-06	22.03
rs78069175	17	66132894	T	C	-0.32608	0.0706999	3.98499E-06	21.27
rs8047170	16	810258	G	A	0.273777	0.0567885	1.42843E-06	23.24
rs8050812	16	30374516	C	T	-0.117763	0.0172855	9.57275E-12	46.40

AA2S,Ascorbic Acid 2-Sulfate

CHR, chromosome; POS, position; EA, effect allele; OA, other allele; EAF, effect allele frequency.

Supplementary Table S6: Heterogeneity and pleiotropy in MR analyses.

EXPOSURE	OUTCOME	METHOD	HETEROGENEITY		HORIZONTAL PLEIOTROPY			
					MR-EGGER REGRESSION			MR-PRESSO
			Q	Q P-VALUE	EGGER INTERCEPT	SE	P-VALUE	GLOBAL TEST P-VALUE
IgD-CD24-AC	IS	MR Egger	17.39	0.36	0.102	0.026	0.699	0.426
IgD-CD24-AC	IS	Inverse variance weighted	17.56	0.42				
IS	IgD-CD24-AC	MR Egger	14.62	0.33	-0.031	0.021	0.166	0.303
IS	IgD-CD24-AC	Inverse variance weighted	17.04	0.25				
AA2S	IS	MR Egger	20.6	0.66	-0.004	0.014	0.799	0.816
AA2S	IS	Inverse variance weighted	20.67	0.71				
IgD-CD24-AC	AA2S	MR Egger	6.62	0.98	-0.005	0.011	0.64	0.993
IgD-CD24-AC	AA2S	Inverse variance weighted	6.85	0.99				

IgD- CD24- AC,IgD- CD24- B Cell absolute count， AA2S,Ascorbic Acid 2-Sulfate
MR, Mendelian Randomization; MR-PRESSO, MR-Pleiotropy Residual Sum and Outlier method.

Supplementary Figure S1: Forest plot of causal effect of immune cells on IS

exposure	method	nsnp	pval	OR(95%CI)	outcome
IgD− CD24− AC	Inverse variance weighted	18	0.0014	1.2162 (1.0789−1.3711)	ischemic stroke
CD8 on CD28+ CD45RA− CD8br	Inverse variance weighted	14	0.0497	1.1563 (1.0002−1.3368)	ischemic stroke
CD127 on CD45RA+ CD4+	Inverse variance weighted	22	0.0086	1.1313 (1.0318−1.2405)	ischemic stroke
CM DN (CD4−CD8−) %DN	Inverse variance weighted	16	0.0188	1.1296 (1.0204−1.2505)	ischemic stroke
IgD− CD24− %lymphocyte	Inverse variance weighted	24	0.0290	1.1282 (1.0124−1.2572)	ischemic stroke
CD20− CD38− %lymphocyte	Inverse variance weighted	23	0.0280	1.1131 (1.0116−1.2248)	ischemic stroke
CD4 on EM CD4+	Inverse variance weighted	19	0.0237	1.1044 (1.0133−1.2037)	ischemic stroke
BAFF−R on IgD− CD24−	Inverse variance weighted	22	0.0142	1.0959 (1.0185−1.1792)	ischemic stroke
BAFF−R on IgD+ CD24+	Inverse variance weighted	18	0.0056	1.0917 (1.0260−1.1616)	ischemic stroke
BAFF−R on IgD+	Inverse variance weighted	24	0.0070	1.0891 (1.0235−1.1588)	ischemic stroke
BAFF−R on IgD+ CD24−	Inverse variance weighted	22	0.0040	1.0873 (1.0270−1.1512)	ischemic stroke
BAFF−R on IgD− CD38−	Inverse variance weighted	17	0.0081	1.0862 (1.0217−1.1548)	ischemic stroke
CD20− CD38− %B cell	Inverse variance weighted	23	0.0351	1.0846 (1.0057−1.1696)	ischemic stroke
BAFF−R on IgD− CD27−	Inverse variance weighted	18	0.0286	1.0840 (1.0085−1.1653)	ischemic stroke
BAFF−R on CD24+ CD27+	Inverse variance weighted	19	0.0104	1.0826 (1.0188−1.1503)	ischemic stroke
BAFF−R on B cell	Inverse variance weighted	21	0.0194	1.0810 (1.0127−1.1540)	ischemic stroke
BAFF−R on IgD+ CD38dim	Inverse variance weighted	25	0.0025	1.0798 (1.0274−1.1350)	ischemic stroke
BAFF−R on naive−mature B cell	Inverse variance weighted	23	0.0152	1.0786 (1.0147−1.1467)	ischemic stroke
HLA DR++ monocyte AC	Inverse variance weighted	23	0.0458	1.0759 (1.0013−1.1560)	ischemic stroke
FSC−A on T cell	Inverse variance weighted	22	0.0262	1.0743 (1.0085−1.1443)	ischemic stroke
BAFF−R on IgD+ CD38−	Inverse variance weighted	22	0.0388	1.0726 (1.0036−1.1464)	ischemic stroke
CD11c on myeloid DC	Inverse variance weighted	30	0.0392	1.0689 (1.0033−1.1388)	ischemic stroke
BAFF−R on unsw mem	Inverse variance weighted	24	0.0383	1.0599 (1.0031−1.1199)	ischemic stroke
FSC−A on HLA DR+ T cell	Inverse variance weighted	18	0.0328	1.0548 (1.0044−1.1077)	ischemic stroke
CD16−CD56 on HLA DR+ NK	Inverse variance weighted	24	0.0219	0.9397 (0.8910−0.9910)	ischemic stroke
FSC−A on HLA DR+ NK	Inverse variance weighted	20	0.0342	0.9313 (0.8720−0.9947)	ischemic stroke
CD62L− DC %DC	Inverse variance weighted	22	0.0274	0.9233 (0.8602−0.9912)	ischemic stroke
CD24 on memory B cell	Inverse variance weighted	31	0.0035	0.9171 (0.8654−0.9719)	ischemic stroke
IgD− CD38dim AC	Inverse variance weighted	17	0.0471	0.8642 (0.7483−0.9982)	ischemic stroke
CD28− CD8br %T cell	Inverse variance weighted	16	0.0112	0.7873 (0.6544−0.9472)	ischemic stroke

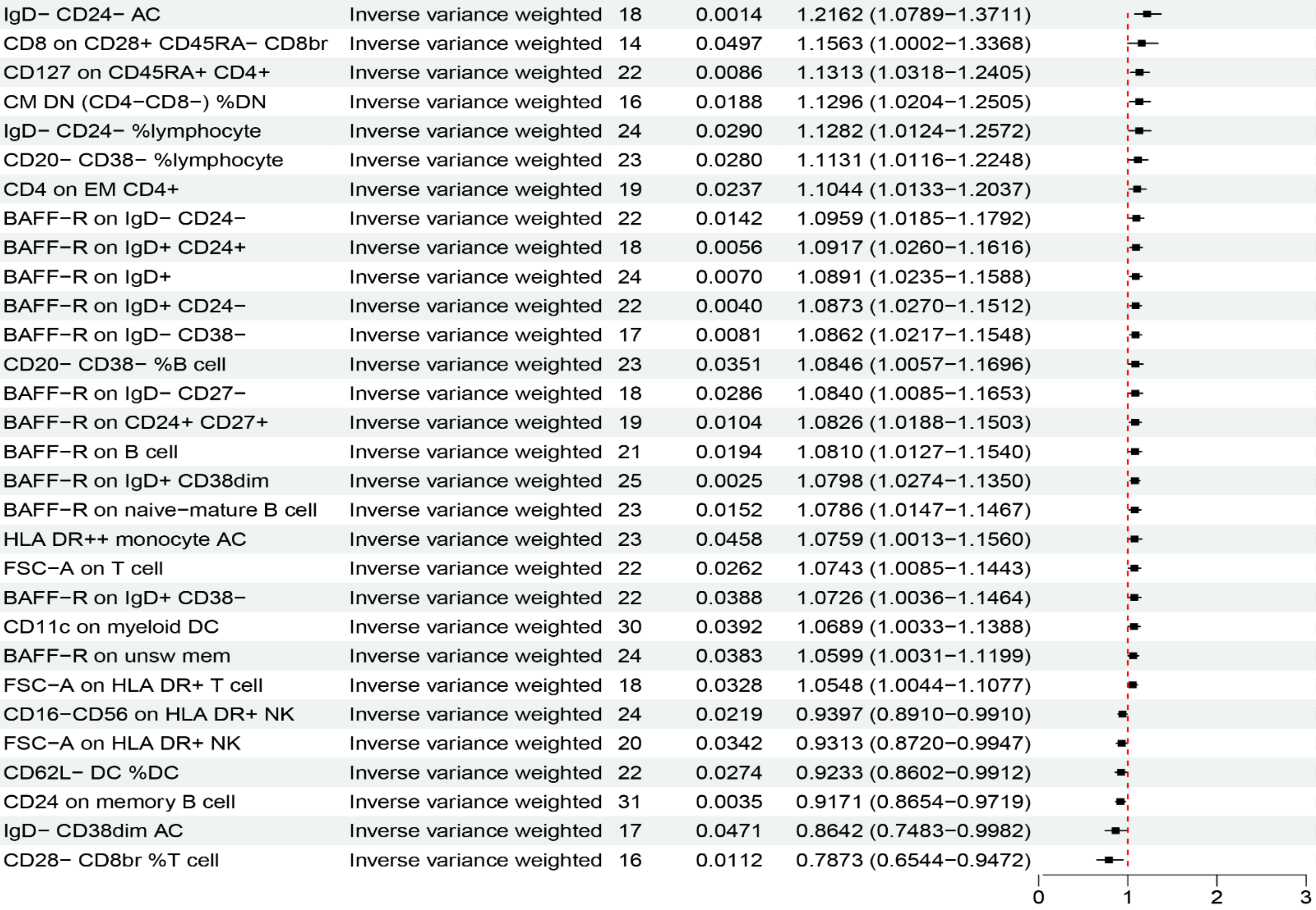

Supplementary Figure S2: Forest plot of causal effect of metabolites on IS

exposure	method	nsnp	pval	OR(95%CI)	outcome
Glutamine conjugate of C6H10O2 (2) levels	Inverse variance weighted	16	0.0208	1.3628 (1.0482−1.7717)	ischemic stroke
3−methoxycatechol sulfate (2) levels	Inverse variance weighted	22	0.0033	1.3434 (1.1030−1.6362)	ischemic stroke
X−24243 levels	Inverse variance weighted	20	0.0253	1.2676 (1.0297−1.5605)	ischemic stroke
3−methoxytyramine sulfate levels	Inverse variance weighted	20	0.0106	1.2497 (1.0533−1.4828)	ischemic stroke
Chiro−inositol levels	Inverse variance weighted	14	0.0397	1.2361 (1.0100−1.5129)	ischemic stroke
X−21285 levels	Inverse variance weighted	30	0.0023	1.2264 (1.0754−1.3986)	ischemic stroke
Cysteinylglycine to taurine ratio	Inverse variance weighted	31	0.0461	1.1849 (1.0030−1.3998)	ischemic stroke
Carotene diol (3) levels	Inverse variance weighted	27	0.0393	1.1823 (1.0082−1.3863)	ischemic stroke
Spermidine to adenosine 5'−diphosphate (ADP) ratio	Inverse variance weighted	23	0.0307	1.1808 (1.0156−1.3730)	ischemic stroke
Ascorbic acid 2−sulfate levels	Inverse variance weighted	26	0.0208	1.1404 (1.0202−1.2748)	ischemic stroke
Androsterone sulfate levels	Inverse variance weighted	36	0.0299	1.0839 (1.0079−1.1657)	ischemic stroke
X−18887 levels	Inverse variance weighted	19	0.6365	1.0831 (0.7777−1.5085)	ischemic stroke
N−acetyl−aspartyl−glutamate (naag) levels	Inverse variance weighted	19	0.5948	1.0296 (0.9247−1.1464)	ischemic stroke
Adenosine 5'−monophosphate (AMP) to tryptophan ratio	Inverse variance weighted	27	0.6417	0.9547 (0.7854−1.1605)	ischemic stroke
Orotidine levels	Inverse variance weighted	28	0.0482	0.9492 (0.9014−0.9996)	ischemic stroke
Adenosine 5'−monophosphate (AMP) to urate ratio	Inverse variance weighted	27	0.5745	0.9398 (0.7568−1.1671)	ischemic stroke
Glutamine levels	Inverse variance weighted	26	0.2846	0.9112 (0.7686−1.0804)	ischemic stroke
Tauro−beta−muricholate levels	Inverse variance weighted	26	0.0412	0.9031 (0.8189−0.9959)	ischemic stroke
N−acetyl−beta−alanine levels	Inverse variance weighted	27	0.0071	0.8748 (0.7936−0.9643)	ischemic stroke
X−21471 levels	Inverse variance weighted	27	0.0196	0.8674 (0.7697−0.9775)	ischemic stroke
Adenosine 5'−monophosphate (AMP) to threonine ratio	Inverse variance weighted	25	0.0294	0.8417 (0.7209−0.9829)	ischemic stroke
1−stearoyl−2−oleoyl−GPI (18:0/18:1) levels	Inverse variance weighted	24	0.0399	0.8375 (0.7071−0.9919)	ischemic stroke
Suberate (C8−DC) levels	Inverse variance weighted	27	0.0206	0.8360 (0.7184−0.9729)	ischemic stroke
Furaneol sulfate levels	Inverse variance weighted	15	0.0446	0.8205 (0.6765−0.9952)	ischemic stroke
Bilirubin (Z,Z) to taurocholate ratio	Inverse variance weighted	19	0.0382	0.8158 (0.6729−0.9890)	ischemic stroke
X−24585 levels	Inverse variance weighted	18	0.0361	0.8132 (0.6701−0.9867)	ischemic stroke
X−23587 levels	Inverse variance weighted	25	0.0369	0.8074 (0.6605−0.9871)	ischemic stroke
Quinolinate levels	Inverse variance weighted	20	0.0327	0.8006 (0.6528−0.9819)	ischemic stroke
X−11843 levels	Inverse variance weighted	23	0.0218	0.7984 (0.6586−0.9678)	ischemic stroke
Gamma−glutamylhistidine levels	Inverse variance weighted	22	0.0074	0.7971 (0.6752−0.9411)	ischemic stroke
1−(1−enyl−palmitoyl)−2−arachidonoyl−GPE (p−16:0/20:4) levels	Inverse variance weighted	20	0.0258	0.7914 (0.6442−0.9722)	ischemic stroke
X−11372 levels	Inverse variance weighted	25	0.0108	0.7905 (0.6599−0.9471)	ischemic stroke
5−hydroxyindole sulfate levels	Inverse variance weighted	18	0.0160	0.7746 (0.6292−0.9536)	ischemic stroke
Phenylalanine to tyrosine ratio	Inverse variance weighted	19	0.0108	0.7746 (0.6365−0.9426)	ischemic stroke
1−stearoyl−2−oleoyl−GPS (18:0/18:1) levels	Inverse variance weighted	20	0.0087	0.7465 (0.6001−0.9287)	ischemic stroke
Laurate (12:0) levels	Inverse variance weighted	19	0.0089	0.6905 (0.5231−0.9114)	ischemic stroke

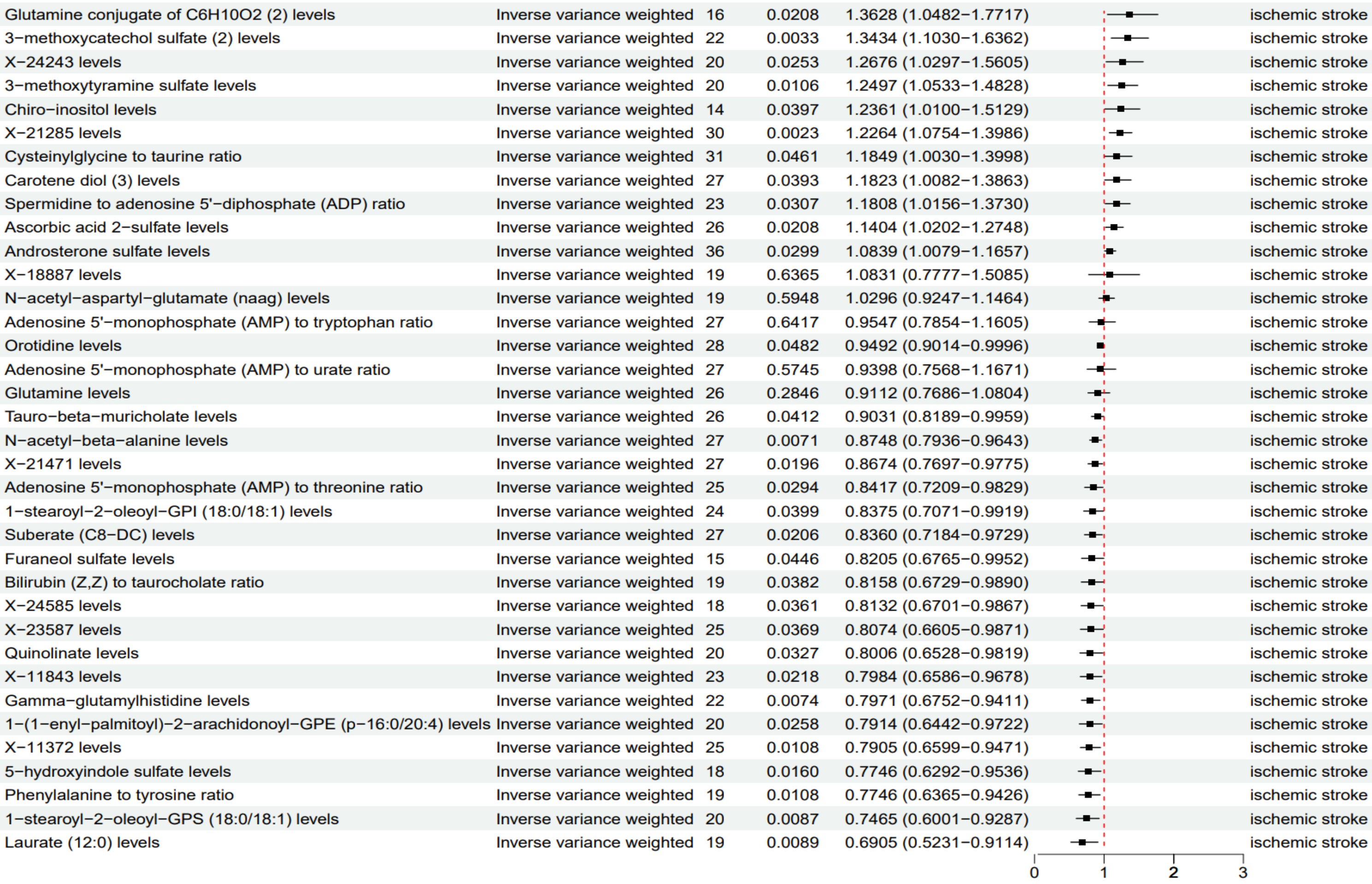

Supplementary Figure S3: leave one out sensitivity analysis for IgD- D24- AC on IS

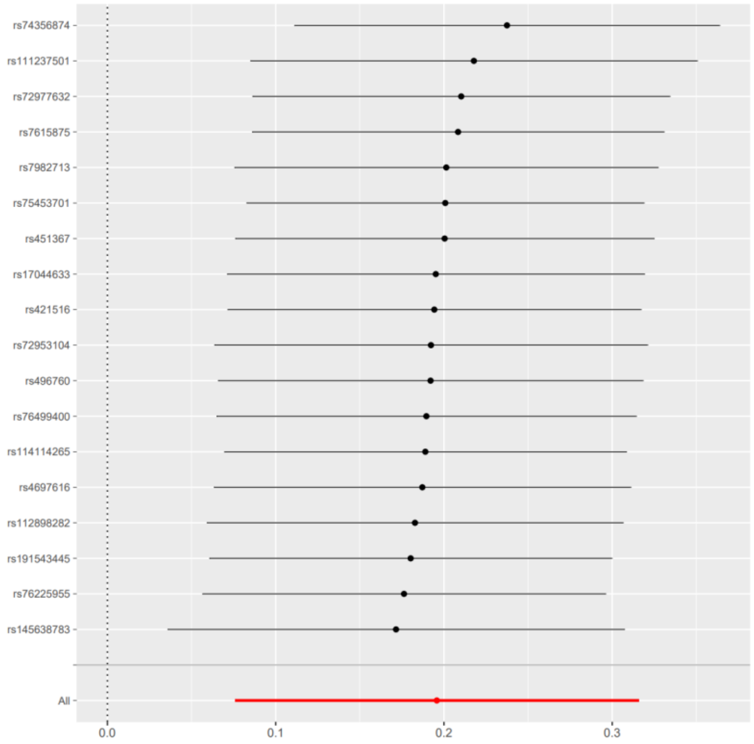

Supplementary Figure S4: funnel plot of IgD- D24- AC on IS

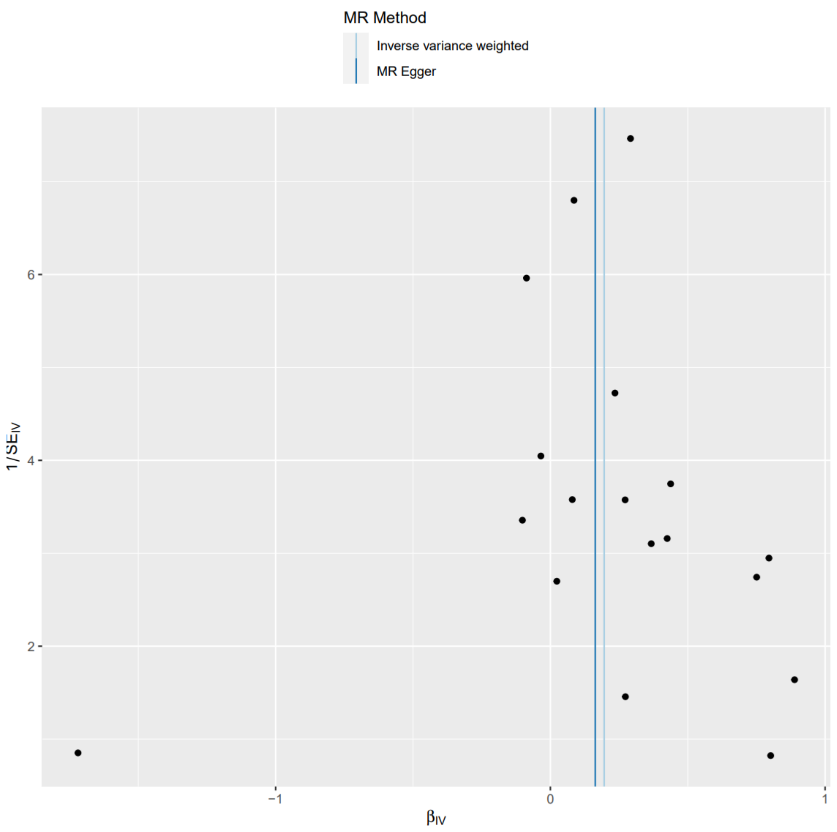

Supplementary Figure S5: Forest plot of causal effect of AA2S on total IS risk

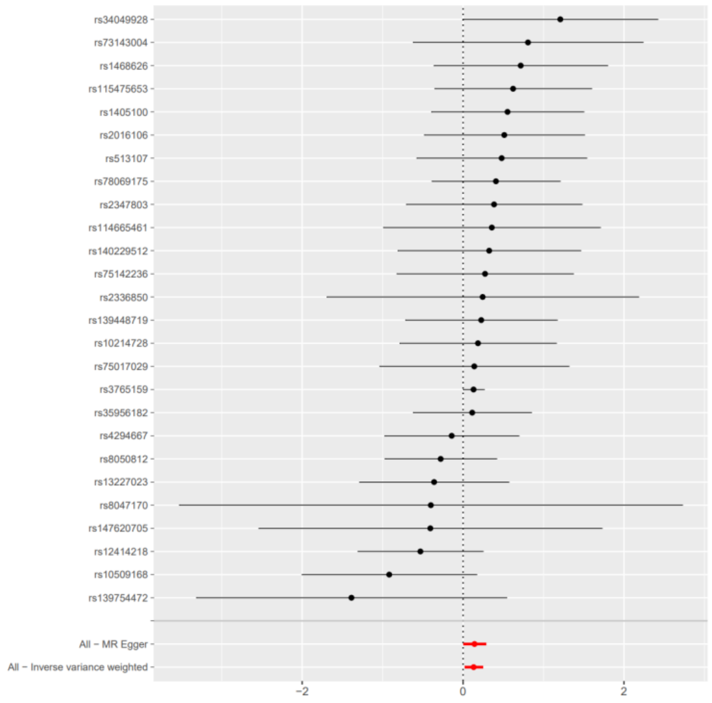

Supplementary Figure S6: Forest plot of causal effect of IgD- D24- AC on AA2S

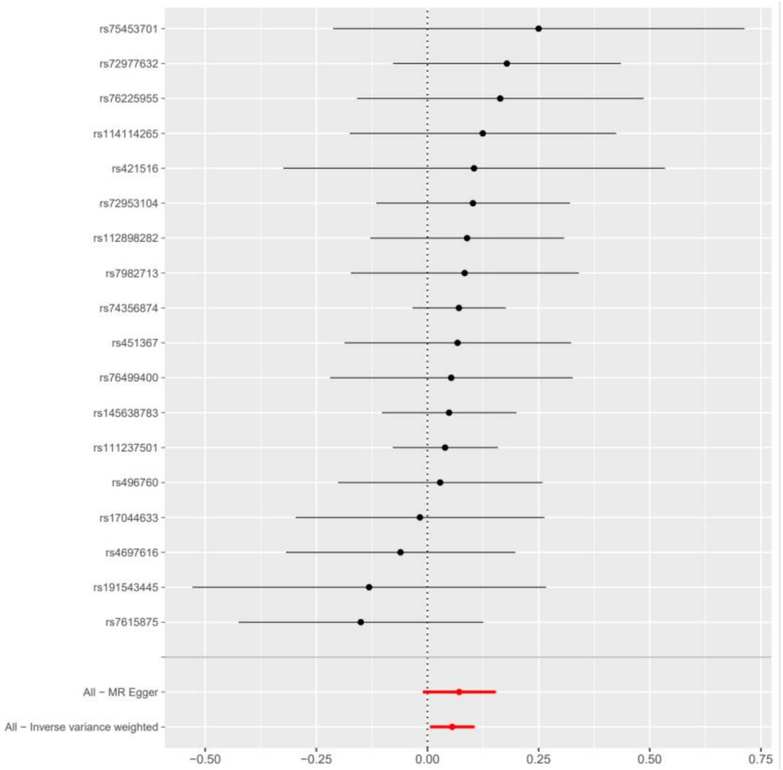

Supplement: Supplementary file 1 [file Data_Sheet_1.PDF]
